# Integrative cross-tissue transcriptome-wide association and metabolomic analysis reveals novel genetic risk loci for aortic aneurysm

**DOI:** 10.3389/fnut.2026.1795141

**Published:** 2026-08-24

**Authors:** Hanxi Wang, Junjie Cheng, Jiali Yao, Wentao Jin, Wei Li

**Affiliations:** 1 Department of Anesthesiology, The Fourth Affiliated Hospital of School of Medicine, and International School of Medicine, International Institutes of Medicine, Zhejiang University, Yiwu, China; 2 Department of Nephrology, Center for Regeneration and Aging Medicine, The Fourth Affiliated Hospital of School of Medicine, and International School of Medicine, International Institutes of Medicine, Zhejiang University, Yiwu, China; 3 Department of Critical Care Medicine, Jinhua Hospital Affiliated to Zhejiang University, Jinhua, Zhejiang, China; 4 The Fourth Affiliated Hospital, Zhejiang University School of Medicine, Futian Campus, Yiwu, China; 5 Department of Vascular Surgery, The Second Hospital of Yinzhou District, Ningbo, China

**Keywords:** aortic aneurysm, colocalization, cross-tissue TWAS, MAGMA, Mendelian randomization

## Abstract

**Background:**

Aortic aneurysm (AA) is a life-threatening cardiovascular condition with a strong genetic component, however, its molecular mechanisms remain poorly understood. Although genome-wide association studies (GWAS) have identified numerous risk loci, most prior studies have investigated genetic and metabolic factors separately, leaving the causal pathways from genetic variants to disease largely unexplored.

**Methods:**

We established an integrative framework combining cross-tissue transcriptome-wide association studies (TWAS) with metabolomic mediation analysis. First, we integrated GWAS data from FinnGen R12 with multi-tissue expression quantitative trait loci (eQTL) data from Genotype-Tissue Expression Project (GTEx) V8, then performed cross-tissue TWAS using the Unified Test for MOlecular SignaTures (UTMOST) and single-tissue validation with the Functional Summary-based Imputation (FUSION) to prioritize susceptibility genes. Second, we applied Mendelian randomization (MR), colocalization, and Fine-mapping Of CaUsal gene Sets (FOCUS) to assess causality and identify high-confidence genes. Third, we performed metabolite mediation analysis to uncover metabolic pathways linking genetic variants to disease risk. Finally, we validated key findings in mouse models of thoracic aortic aneurysm (TAA) and abdominal aortic aneurysm (AAA) using Quantitative Real-Time Reverse Transcription Polymerase Chain Reaction (RT-qPCR) and Western blotting.

**Results:**

We identified multiple novel susceptibility genes for AA and its subtypes. Key genes included ADH family members (*ADH1A, ADH1B, ADH4, ADH6*) and *ZNF827*, which showed cross-subtype associations with strong colocalization evidence in vascular tissues. Metabolite mediation analysis revealed significant pathways involving N-acetylphenylalanine and methionine sulfoxide. Functional enrichment revealed distinct biological mechanisms: AA and AAA were primarily associated with metabolic pathways, whereas TAA-related genes were enriched in developmental and contractile processes. PheWAS indicated no significant off-target associations. Critically, experimental validation in mouse models confirmed significant upregulation of *ZNF827* in TAA and *ADH6* in AAA at both mRNA and protein levels, corroborating the genetic predictions.

**Conclusion:**

This integrated cross-omics analysis identifies novel genetic loci and, crucially, uncovers specific nutrient-related metabolic pathways that mediate genetic risk. These findings provide a mechanistic basis for future nutritional and metabolic intervention studies in AA and its subtypes.

## 1 Introduction

Aortic aneurysm (AA), a life-threatening cardiovascular disease, is characterized by pathological dilation of the aortic wall (1). Based on anatomical location, AA is typically classified into two types: thoracic aortic aneurysm (TAA) and abdominal aortic aneurysm (AAA) (2). However, concurrent lesions involving both aortic regions may occur. As the second most prevalent aortic pathology following atherosclerosis, AA features both insidious progression and acute rupture risk, creating substantial clinical challenges (3). Ranking as the 15th leading cause of mortality in individuals ≥55 years and 19th globally, AA imposes substantial burden on worldwide healthcare systems (4). Although surgical management has advanced, the molecular pathogenesis of AA remains elusive, hindering the development of targeted therapeutics.

Epidemiological studies have firmly established associations between AA and modifiable risk factors such as smoking, hypertension, and atherosclerotic disease (4, 5). However, genetic predisposition is increasingly recognized as a major independent risk determinant. Twin- and family-based studies estimate that additive genetic factors account for approximately 77% of the risk variance for AAA and up to 70% for TAA (6, 7). Genome-wide association studies (GWAS) have identified 141 independent risk loci for AAA, including 97 newly discovered loci (8). Separately, 21 risk loci were associated with thoracic aortic aneurysm and dissection (TAAD), 17 of which represent new discoveries (9). These loci implicate biological pathways related to extracellular matrix remodeling, inflammatory responses, and vascular smooth muscle function. Nevertheless, a substantial portion of disease-associated loci identified through GWAS reside in non-coding genomic segments, complicating the evaluation of their biological relevance (10). Furthermore, the complexity of linkage disequilibrium (LD) within risk regions often obscures true causal variants, thereby limiting the translational potential of GWAS findings (11).

To address these limitations, transcriptome-wide association studies (TWAS) have emerged as a powerful approach that integrates expression quantitative trait loci (eQTL) data with GWAS summary statistics, enabling the prioritization of candidate genes and the prediction of disease-relevant expression patterns (12). However, traditional single-tissue TWAS approaches fail to adequately capture the extensive gene expression patterns and regulatory characteristics in multisystem disorders like AA. These pathologies likely arise from coordinated gene expression across vascular, immune, and connective tissue systems. To overcome these constraints, cross-tissue TWAS frameworks like the Unified Test for Molecular Signatures (UTMOST) have been developed to perform gene-trait association analyses across multiple tissues (13). This method improves the accuracy and effectiveness of the imputation model by imposing a “group-lasso penalty,” while encouraging cross organizational sharing of eQTLs and maintaining tissue-specific eQTLs with strong effects. This framework has demonstrated efficacy in identifying novel susceptibility genes for systemic disorders including chronic obstructive pulmonary disease, migraines, and sleep apnea (14–16).

Although previous GWAS and TWAS have identified numerous risk loci for AA and its subtypes, most studies have investigated genetic associations and metabolic alterations independently, without integrating them into a unified framework. More importantly, the extent to which these genetic risks are mediated by modifiable nutritional and metabolic factors remains largely unexplored. Given that AA pathogenesis involves pathways such as retinol (vitamin A) metabolism, lipid handling, and amino acid catabolism—all of which are directly influenced by dietary intake and nutritional status—there is a compelling need to bridge the gap between genetic susceptibility and nutritional metabolism. To fill this gap, we established an integrative cross-tissue transcriptome-wide and metabolomic analysis framework. We combined multi-tissue eQTL data from Genotype-Tissue Expression Project (GTEx) V8 with GWAS summary statistics from FinnGen R12, and further incorporated metabolite mediation analysis, Mendelian randomization (MR), colocalization, and fine-mapping. Using this integrated approach, we systematically prioritized novel susceptibility genes and dissected the metabolic pathways (e.g., amino acid derivatives, phytosterols, retinol metabolism) through which they may exert their effects. This unified, nutrition-informed approach allowed identification of both genetic risk loci and intermediate metabolic mediators, thereby providing a more mechanistic understanding of AA and its subtypes than previously possible. Finally, we experimentally validated key findings in mouse models of TAA and AAA, confirming differential expression of *Zfp827* and *Adh6a* at both mRNA and protein levels.

## 2 Methods

### 2.1 Study design

**Figure 1:**
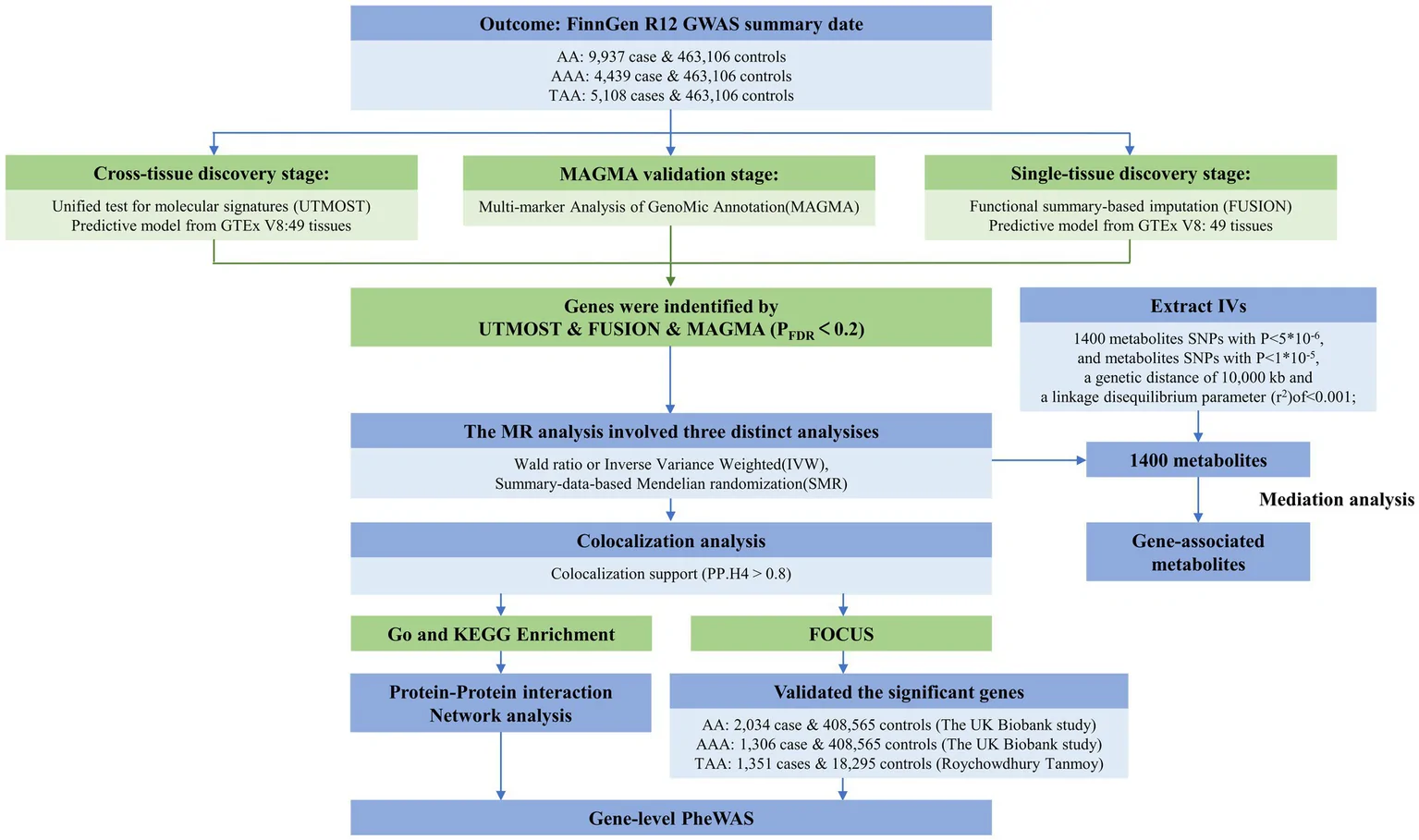
The flowchart of this study.

This study employed a multi-step analytical framework to identify novel susceptibility genes for AA and its subtypes (AAA and TAA) through cross-tissue TWAS. The analytical workflow integrated GWAS summary statistics, eQTL data, and functional annotations to systematically prioritize candidate genes and elucidate underlying biological pathways. A schematic overview of the analysis pipeline is provided in Figure 1.

### 2.2 eQTL data sources

Tissue-specific eQTL data were derived from the GTEx V8 (17, 18), encompassing 49 human tissues collected from 838 post-mortem donors.^1^ This resource provides multi-omics data including gene expression profiles, genetic variations, co-expression networks, and eQTL analyses. It serves as a foundational platform for disease mechanism studies, gene functional annotation, and therapeutic discovery.

### 2.3 GWAS outcomes data sources

The primary analysis of AA, AAA, and TAA outcomes is based on the summary statistical data from FinnGen R12 (19),^2^ which includes 9,937 cases of AA, 4,439 cases of AAA, 5,108 cases of TAA, and 463,106 controls of European ancestry.

### 2.4 Cross-tissue TWAS analysis

We conducted cross-tissue TWAS using the UTMOST^3^ approach to estimate overall gene-trait associations at the organismal level. This strategy combines tissue-specific eQTL effects with GWAS summary statistics, enabling the identification of more genes within tissues, thereby improving imputation accuracy and demonstrating rich trait heritability (13). The Generalized Berk-Jones (GBJ) test was then applied to integrate gene-trait associations by utilizing covariance obtained from single-tissue data. After applying the false discovery rate (FDR) correction, genes with FDR < 0.2 are considered statistically significant.

### 2.5 Single-tissue TWAS analysis

The Functional Summary-based Imputation (FUSION) method^4^ was employed to validate gene-trait associations in single tissues. We integrated GWAS summary statistics with pre-computed tissue-specific gene expression prediction weights for GTEx V8, obtained from the FUSION website (12). These weights were derived using multiple prediction models (e.g., BLUP, BSLMM, LASSO, Elastic Net, and Top1), with the optimal model selected via cross-validation based on predictive accuracy. To account for linkage disequilibrium (LD) among SNPs within each gene model, we used the 1,000 Genomes Project European reference panel to estimate the LD correlation matrix, which was then incorporated into the test statistics to correct for correlated SNP effects. The resulting gene-level Z-scores were used to evaluate the association between predicted gene expression and disease risk. Genes with FDR < 0.2 in at least one tissue were considered significant.

### 2.6 Gene-level association

We conducted gene-based association analysis using Multi-marker Analysis of Genomic Annotation (MAGMA) software (version 1.08) to systematically assess the genetic relationships between genes and target phenotypes. MAGMA combines the effects of multiple SNPs within genes to calculate gene-level association scores, providing a comprehensive analysis of how genetic variations contribute to phenotypes (20, 21). This method’s key advantage is its ability to detect the cumulative effects of multiple low-impact SNPs, thereby enhancing the identification of complex trait genetic architectures. Finally, integrating results from MAGMA, UTMOST, and FUSION analyses, we identified robust candidate genes that passed FDR correction (*p* < 0.2) across multiple methods. For detailed MAGMA algorithms, parameter settings, and methodological descriptions, please consult the original publications (22).

### 2.7 MR analysis

We performed MR analysis using the “TwoSampleMR” R package (version 0.6.6) (23). In this analysis, cis-eQTL SNPs served as instrumental variables (IVs), with gene expression as the exposure and GWAS data as the outcome. To ensure the independence of IVs, genome-wide significant SNPs (*p* < 5 × 10^−8^) are typically selected. However, since such *p*-values cannot be extracted from the tissues in GTEx V8 and the sample size for each tissue is relatively small, we did not apply the p-value threshold. Nevertheless, we still performed LD clumping (r^2^ < 0.001,window = 10,000 kb) in our analysis to maintain the independence of the IVs. For analyses with a single IV, we applied the Wald ratio method. For analyses with multiple IVs, we employed the inverse-variance weighted (IVW) method, with statistical significance set at *p* < 0.05. This approach effectively evaluates potential causal relationships between gene expression levels and AA risk.

To address limitations in heterogeneity testing for single-SNP MR analyses, we implemented Summary-data-based MR (SMR) analysis^5^ for causal effect estimation. Using the Heterogeneity in Dependent Instruments (HEIDI) test, we differentiated between pleiotropic and linkage models (24). Results with HEIDI *p*-value < 0.05 were considered to exhibit potential pleiotropy. Such results were then excluded to mitigate pleiotropic effects. We set the SMR significance threshold at *p* < 0.05 to identify phenotype-associated genes with potential pathogenic effects and to further explore gene-trait causal relationships (25).

### 2.8 Colocalization analysis

Subsequently, we performed Bayesian colocalization analysis using the “coloc” R package to assess whether GWAS and eQTL signals share causal genetic variants (26). The analysis evaluates five competing hypotheses using posterior probability (PP), with PP. H4 as the primary colocalization evidence metric (27). Following established consensus, we considered PP. H4 > 0.8 as the significance threshold, suggesting shared genetic regulation between the GWAS trait and gene expression. This finding implies a functional link between GWAS signals and eQTL loci, offering insights into the regulatory mechanisms of trait-associated genes.

### 2.9 Mediation analysis of metabolites

Derived from the Canadian Longitudinal Study on Aging (CLSA) cohort, our metabolite dataset includes 8,299 unrelated individuals of European ancestry, with summary statistics available for 1,091 plasma metabolites and 309 metabolite ratios (28). This comprehensive dataset covers different biochemical categories, including lipids, amino acids, xenobiotics, nucleotides, cofactors and vitamins, carbohydrates, peptides, and energy-related metabolites. Analysis of all 1,400 metabolites revealed exhaustive extraction of SNPs meeting the stringent genome-wide significance threshold (*p* < 5 × 10^−8^) was infeasible. To enhance detection sensitivity and capture broader results, we defined instrumental variables (IVs) as SNPs below the significance threshold of 5 × 10^−6^ and 1 × 10^−5^. To minimize confounding from correlated SNPs, we performed LD clumping (r^2^ = 0.001, window = 10,000 kb) on all IVs.

We implemented a two-stage MR framework to evaluate metabolite-mediated pathways linking genome-wide significant genetic variants to AA and its subtypes (AAA and TAA). Stage I estimated causal effects of significant genes on metabolite concentrations (β1). Stage II assessed causal effects of metabolites on AA and its subtypes (AAA and TAA) risk (β2). The mediation proportion equaled (β1 × β2)/β3 with β3 representing total genetic effect on AA and its subtypes (AAA and TAA). The 95% confidence interval (CI) of the proportion of mediation was calculated using the coefficient product method, with significance threshold at two-sided *p* < 0.05.

### 2.10 Fine-mapping of causal gene sets (FOCUS)

To further validate the results of MR and pinpoint causal genes from TWAS signals, we employed FOCUS for fine-mapping analysis. This method utilizes GWAS summary statistics, eQTL prediction weights, and LD as inputs to generate a set of credible output genes associated with observed genomic risk (29). FOCUS models the correlation structure of gene expression predictions to estimate the posterior probability that each gene set contains causal genes. In our analysis, we strictly selected risk-associated genes using a marginal posterior inclusion probability (PIP) > 0.8 and a *p*-value < 5 × 10^−8^.

### 2.11 GO and KEGG pathway analysis

We conducted Gene Ontology (GO) and Kyoto Encyclopedia of Genes and Genomes (KEGG) enrichment analyses to characterize biological functions and pathway features of the target gene set (30). GO enrichment analysis reveals gene functional characteristics from three dimensions, including biological processes, cellular components, and molecular functions. This method identifies significantly enriched functional categories (*p* < 0.05) by statistically comparing the target gene set with the reference genome. KEGG analysis focuses on gene enrichment in metabolic pathways and signal transduction networks. Collectively, these analyses reveal coordinated gene functions within biological processes and metabolic pathways.

### 2.12 Protein–protein interaction (PPI) networks

We conducted a PPI network analysis to explore interactions among target proteins. Using GeneMANIA,^6^ we constructed the PPI network with a minimum interaction confidence threshold of 0.400 (the default setting) and other default parameters (31).

### 2.13 Validation set

We validated the genes identified in the FinnGen R12 results using the UK Biobank cohort^7^ for AA (2,034 cases and 408,565 controls) and AAA (1,306 cases and 408,565 controls), all of European ancestry (32). For TAA, as no corresponding results were available in the UK Biobank, we performed validation and association replication using GWAS summary statistics from Roychowdhury et al. (1,351 cases and 18,295 controls), also restricted to European-ancestry individuals (33).

### 2.14 Phenome-wide association analysis

To evaluate the feasibility of future gene-based drug prediction and assess pleiotropy and adverse effect risks of candidate drug targets, we conducted a phenome-wide association study (PheWAS) using the AstraZeneca PheWAS Portal^8^ (34). Gene expression functioned as exposure, with outcomes comprising 15,500 binary and 1,500 continuous phenotypes from exome sequencing of UK Biobank participants (*n* ≈ 450,000).

### 2.15 Animal models

#### 2.15.1 Mouse TAA model (BAPN+AngII)

Three-week-old male C57BL/6 SPF mice (8–10 g) were acclimatized for 1 week, then stratified randomly into control and TAA groups (*n* = 6 per group), with blinding. The TAA group received sterile water containing 2.5 g/L BAPN (daily water intake recorded, weekly replacement), the control group got standard water. After 28-day modeling, the TAA group subcutaneous implantation of Alzet 1,004 osmotic minipumps (infusing Ang II at 1000 ng/kg/min); the control group got saline-filled pumps. All surgical procedures were performed under anesthesia with appropriate perioperative analgesia. Postoperative health was monitored daily. Mice showing signs of infection, severe weight loss, or those that died prematurely were excluded from the study. After another 28-day, at endpoint, tail-cuff blood pressure and ascending aortic maximum diameter (via Vevo 2,100 echocardiography) were measured. Mice were euthanized, and ascending aorta/aortic arch were collected, fixed in 4% paraformaldehyde or snap-frozen in liquid nitrogen.

#### 2.15.2 Mouse AAA model

The animal model was constructed using the calcium chloride (Calciumchloride, CaCl_2_) induction method. The specific operation steps are as follows: Mice (8 weeks old) were randomly assigned to control or AAA model groups (*n* = 6 per group). After intraperitoneal injection of 1% pentobarbital sodium solution to induce general anesthesia, the experimental animals were fixed on a sterile operating table. Preoperative area preparation included hair removal from the abdomen (using a special depilatory agent) and iodophor disinfection (3 times). A longitudinal incision was made along the midline of the abdomen, and the lower abdominal aorta was fully exposed. The pre-treated 0.5 mol/L CaCl_2_ solution was soaked in sterile gauze and gently wrapped around the target vascular segment, with continuous action for 20 min. Subsequently, the surgical area was thoroughly rinsed with pre-warmed PBS buffer solution, and the abdominal incision was sutured layer by layer using absorbable sutures. The area was then re-disinfected with iodophor. The control group animals were treated with the same operation using an equal amount of normal saline instead of the CaCl_2_ solution, and all other procedures were consistent with the experimental group. Two weeks after modeling, if the modeling was confirmed successful (the abdominal aorta lesion segment expanded to 1.5 times its original size) by ultrasound.

All animal experiments were reviewed and approved by the Animal Ethics Committee of Jinhua Central Hospital (Approval No. AL-JHYY2025).

### 2.16 Quantitative Real-Time Reverse Transcription Polymerase Chain Reaction (RT-qPCR)

Total RNA was extracted from AA tissues using the Eastep® Super Total RNA Extraction Kit according to the manufacturer’s instructions, including an on-column DNase I digestion step to remove genomic DNA. Complementary DNA (cDNA) was synthesized from 1 μg of total RNA using the EasyScript First-Strand cDNA Synthesis SuperMix. RT-qPCR was performed using PerfectStart Green qPCR SuperMix on a CFX96 Real-Time PCR Detection System (Bio-Rad) to quantify the expression of target genes. The thermal cycling conditions were as follows: initial denaturation at 94 °C for 30 s; then 45 cycles of denaturation at 94 °C for 5 s and annealing/extension at 60 °C for 30 s; followed by a melting curve analysis. GAPDH was used as the endogenous reference gene for normalization. Relative gene expression (fold change) was calculated using the 2^–ΔΔCt.^ method. All RT-qPCR assays were performed with three technical replicates for each biological sample.

### 2.17 Western blot

AA tissues were lysed using T-PER Tissue Protein Extraction Reagent (Thermo Fisher Scientific, USA) supplemented with a protease and phosphatase inhibitor cocktail, and protein concentrations were determined using an enhanced BCA protein assay kit (Beyotime Biotechnology, China). Equal amounts of protein (60 μg per lane) were separated by SDS–PAGE and transferred onto PVDF membranes. After blocking with 5% BSA in TBST for 1 h at room temperature, membranes were incubated overnight at 4 °C with primary antibodies against *ZNF827* (1:500, Santa Cruz Biotechnology), *ADH6* (1:1000, Thermo Fisher Scientific), and tubulin (1:10,000, Proteintech). Following incubation with appropriate HRP-conjugated secondary antibodies, protein bands were visualized using an enhanced chemiluminescence substrate (SuperSignal™ West Dura, Thermo Fisher Scientific) and detected by X-ray film. Band intensities were quantified using ImageJ software and normalized to tubulin.

## 3 Results

### 3.1 TWAS analyses in cross-tissue and single tissue

**Figure 2:**
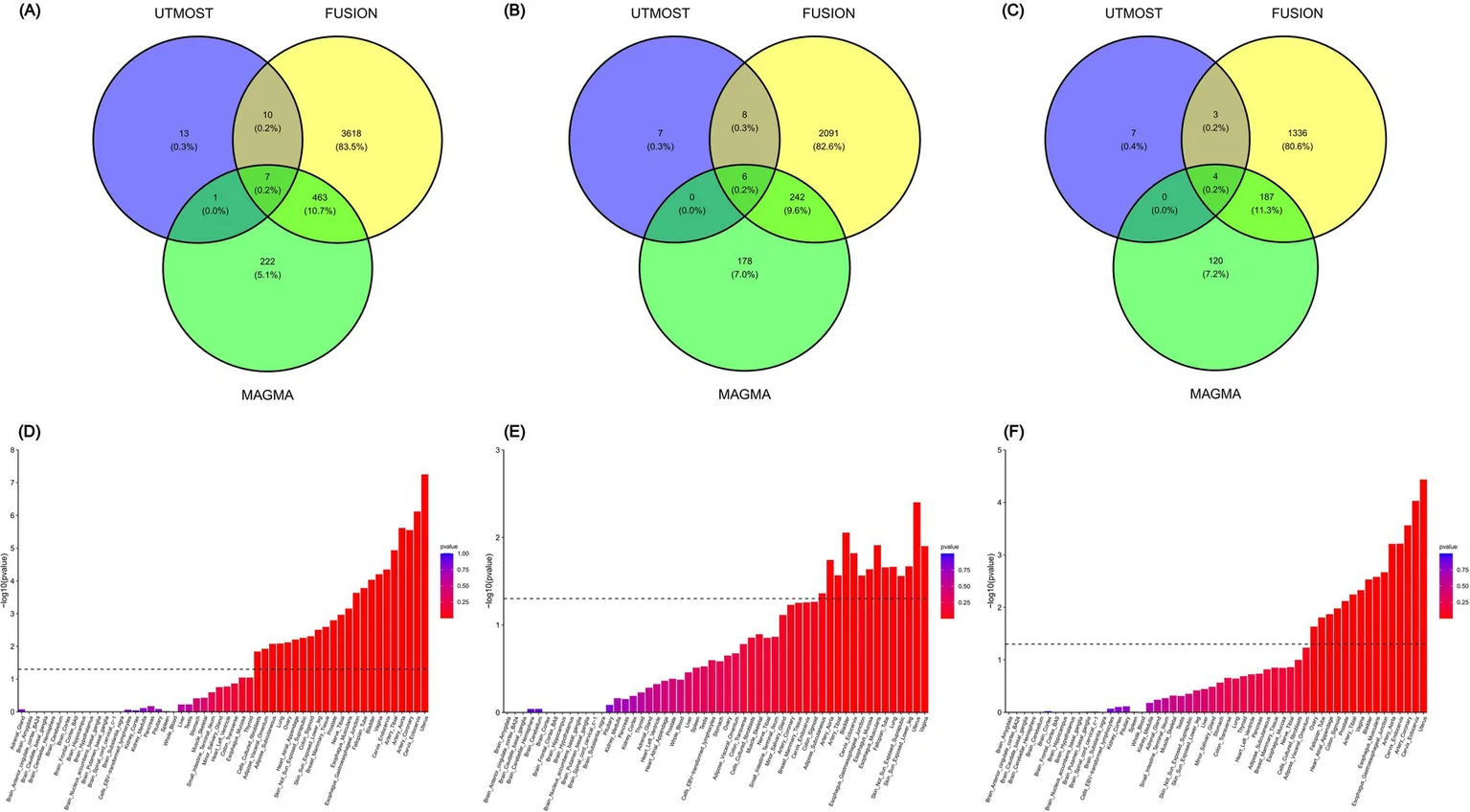
Integration of multi-method gene discovery and tissue enrichment. **(A–C)** Venn diagrams illustrating the overlap of significant genes identified by MAGMA, FUSION, and UTMOST for AA **(A)**, AAA **(B)**, and TAA **(C)** (FDR < 0.2). Numbers in each section indicate the count of unique or shared genes. **(D–F)** Tissue-specific enrichment results for AA **(D)**, AAA **(E)**, and TAA **(F)** based on MAGMA gene-based association analysis.

Cross-tissue TWAS using UTMOST identified significant gene associations for each AA subtype after FDR correction (FDR < 0.2): 31 genes for AA, 21 for AAA, and 14 for TAA (Supplementary Table S1). Single-tissue TWAS using FUSION further detected thousands of significant gene-tissue pairs: 4,098 for AA, 2,347 for AAA, and 1,530 for TAA (Supplementary Tables S2–S4). Integrative analysis of cross-tissue and single-tissue results revealed high-confidence candidate genes meeting strict thresholds in both approaches: 17 for AA, 14 for AAA, and 7 for TAA (Figures 2A–C).

### 3.2 Gene analysis of MAGMA

MAGMA gene-based analysis identified 693 genes for AA, 426 for AAA, and 311 for TAA (Supplementary Tables S5–S7). Tissue-specific enrichment assessment (Figures 2D–F) revealed that AAA and TAA had 14 and 15 significantly enriched tissues, respectively (*p* < 0.05; Figures 2E,F); AA also showed enrichment across multiple tissues (Figure 2D). To enhance robustness, we integrated MAGMA findings with UTMOST cross-tissue and FUSION single-tissue results. This multi-method validation yielded robust gene sets: 7 for AA (*ADH1A, ADH1B, ADH4, ADH6, CCDC74A, GAS2L1, ZNF827*); 6 for AAA (*ADH1A, ADH1B, ADH4, ADH6, ICA1L, WDR12*); and 4 for TAA (*EDNRA, SLC8A1, TNRC6B, ZNF827*) (Figures 2A–C). Notably, the ADH family members were shared between AA and AAA, whereas *ZNF827* appeared in both AA and TAA, suggesting both shared and subtype-specific genetic architectures.

### 3.3 MR, SMR, and colocalization analysis

We performed MR to assess causal relationships between candidate gene expression and each AA subtype, followed by SMR validation and Bayesian colocalization. The number of significant gene-tissue pairs varied across subtypes: 38 for AA, 37 for AAA, and 5 for TAA (*p* < 0.05; Supplementary Table S8). SMR analysis validated causality for 34 AA pairs, 35 AAA pairs, and all 5 TAA pairs. Pleiotropy (HEIDI *p* < 0.05) was detected in 4 AA pairs and 10 AAA pairs but in none of the TAA pairs (Supplementary Table S9).

**Figure 3:**
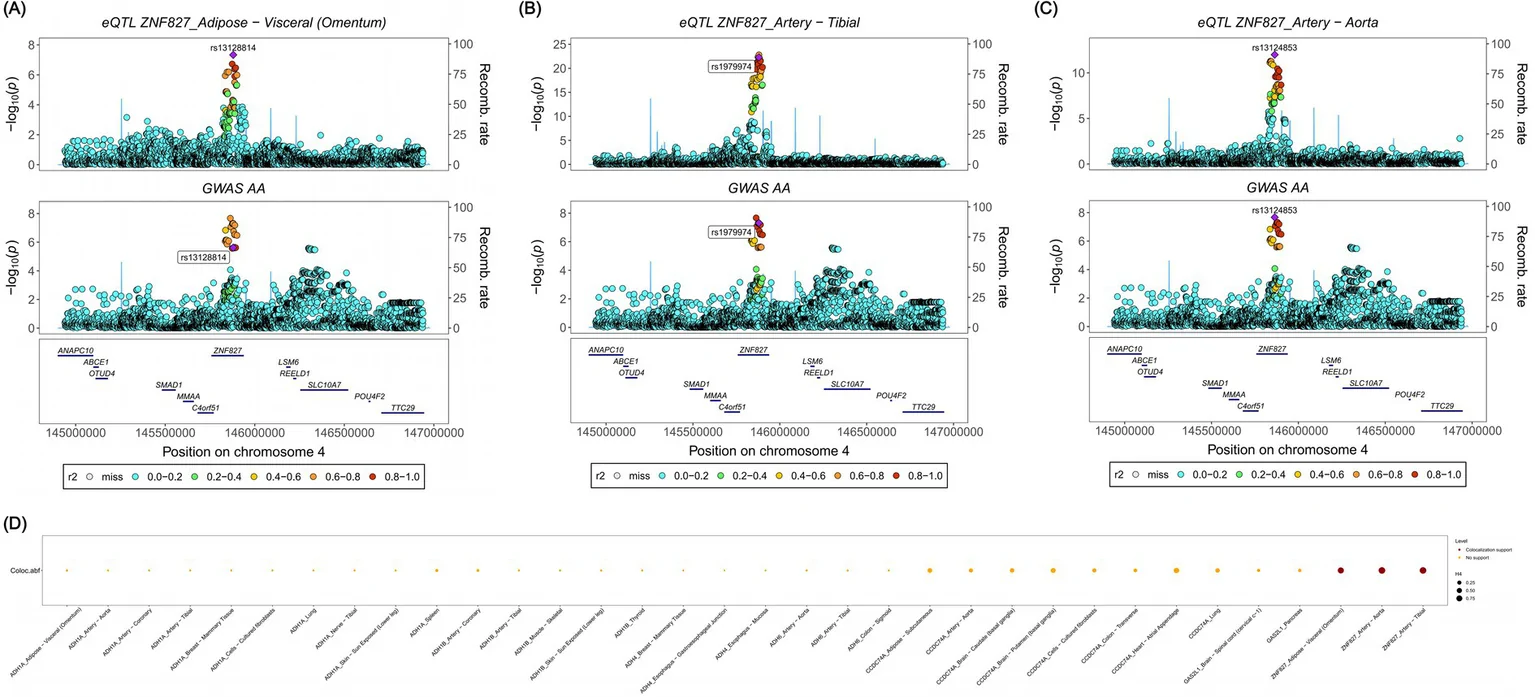
The results of colocalization analysis between candidate genes and AA.

**Figure 4:**
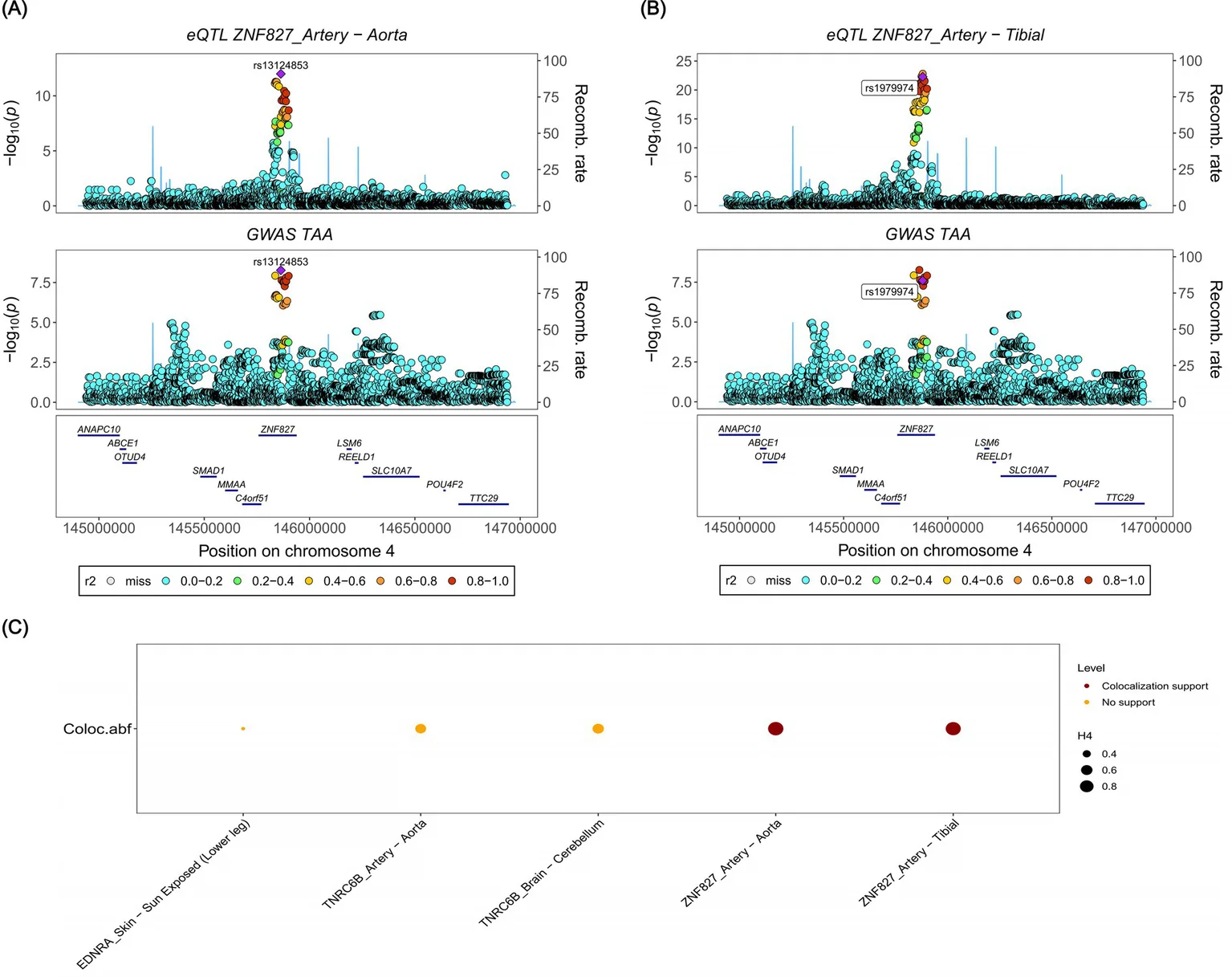
The results of colocalization analysis between candidate genes and TAA.

Colocalization results further distinguished the subtypes. For AA and TAA, *ZNF827* expression shared causal variants with disease risk in vascular tissues: in AA—aorta artery (PP. H4 = 0.991), tibial artery (PP. H4 = 0.977), and visceral adipose tissue (PP. H4 = 0.807); in TAA—aorta artery (PP. H4 = 0.989) and tibial artery (PP. H4 = 0.958) (Supplementary Table S10; Figures 3, 4). These findings implicate *ZNF827*-mediated regulatory mechanisms in the arterial wall as contributors to both AA and TAA pathogenesis. In contrast, despite having the largest number of MR-significant pairs, AAA showed no evidence of colocalization for any candidate gene (all PP. H4 < 0.80), suggesting a distinct regulatory architecture (Supplementary Table S10).

### 3.4 Mediation analysis of metabolites

We performed two-sample MR using two significance thresholds (*p* < 5 × 10^−6^ and *p* < 1 × 10^−5^) to assess causal relationships between 1,400 circulating metabolites and each AA subtype, followed by intersection analysis to identify robust hits. This approach identified 47 metabolites for AA (Supplementary Tables S11–S13) and 46 for AAA (Supplementary Tables S15–S17), but none for TAA (Supplementary Tables S19–S21).

**Figure 5:**
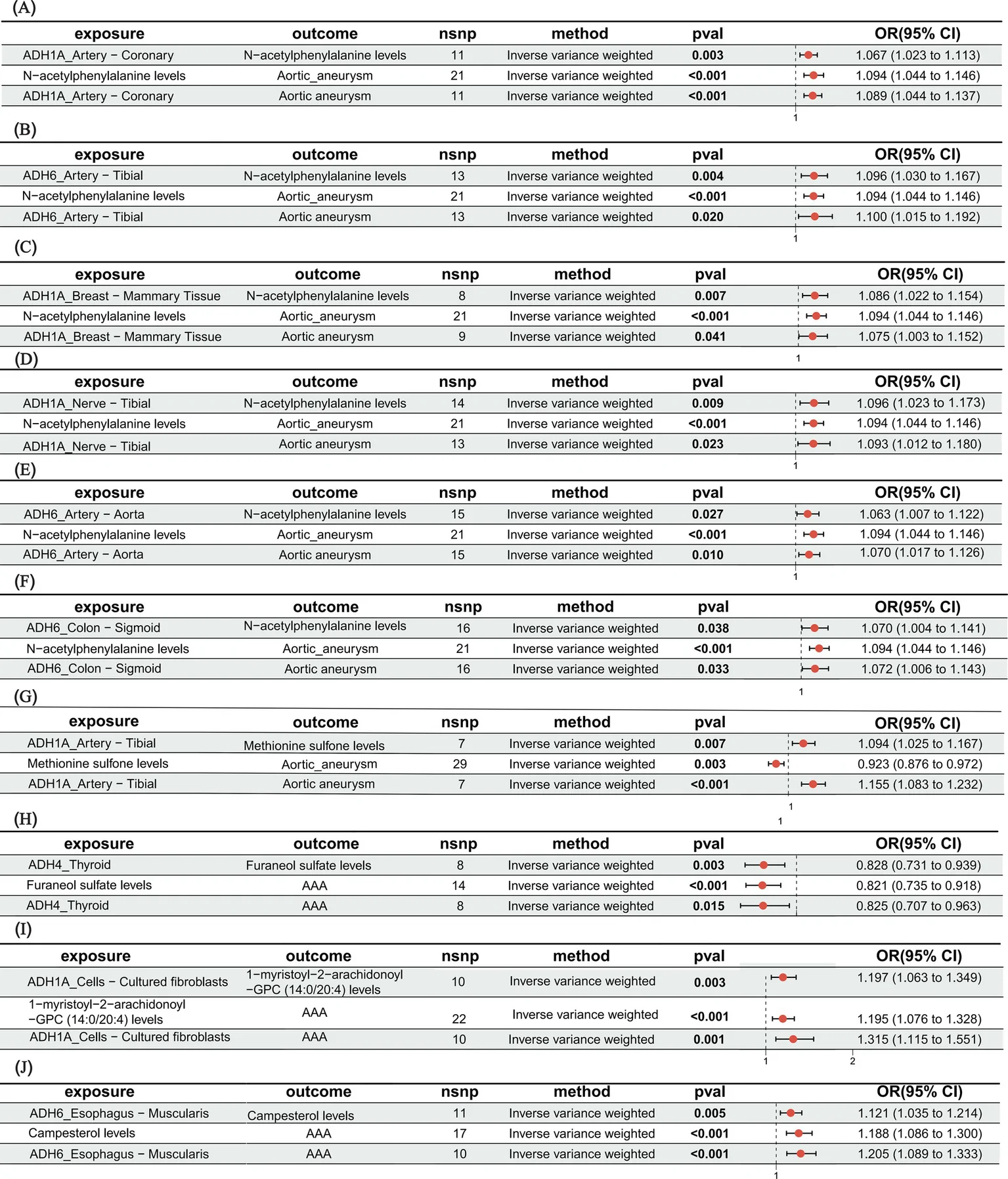
Forest plot of metabolite mediation analysis results.

Mediation analysis of the identified metabolites and candidate genes revealed subtype-specific pathways (Figure 5; Supplementary Tables S14, S18, S22). For AA, N-acetylphenylalanine positively mediated *ADH1A* (in coronary artery, breast tissue, tibial nerve) and *ADH6* (in tibial artery, aorta, sigmoid colon), with mediation proportions of 6.79–10.2% (*p* < 0.05). Conversely, methionine sulfoxide inversely mediated *ADH1A* expression in tibial artery (−5.01, 95% CI: −9.99% to −0.024%, *p* = 4.89 × 10^−2^). For AAA, 1-myristoyl-2-arachidonoyl-GPC (14:0/20:4) positively mediated *ADH1A* in fibroblasts (11.7, 95% CI: 1.33–22.1%, *p* = 2.70 × 10^−2^); campesterol positively mediated *ADH6* in esophageal muscularis (10.6, 95% CI: 0.908–20.3%, *p* = 3.20 × 10^−2^); and furaneol sulfate inversely mediated *ADH4* in thyroid (−19.3, 95% CI: −36% to −2.57%, *p* = 2.37 × 10^−2^). For TAA, despite comprehensive evaluation of all four candidate genes across multiple tissues, no significant mediation effects were detected (all mediation *p*-values > 0.05; Supplementary Table S22).

### 3.5 Focus

To further validate MR findings and pinpoint causal genes, we performed FOCUS fine-mapping within each subtype’s risk loci using a PIP threshold > 0.8. The number and nature of high-confidence causal gene-tissue combinations differed markedly across subtypes: 19 for AA, 8 for AAA, and 8 for TAA (Supplementary Table S23).

**Figure 6:**
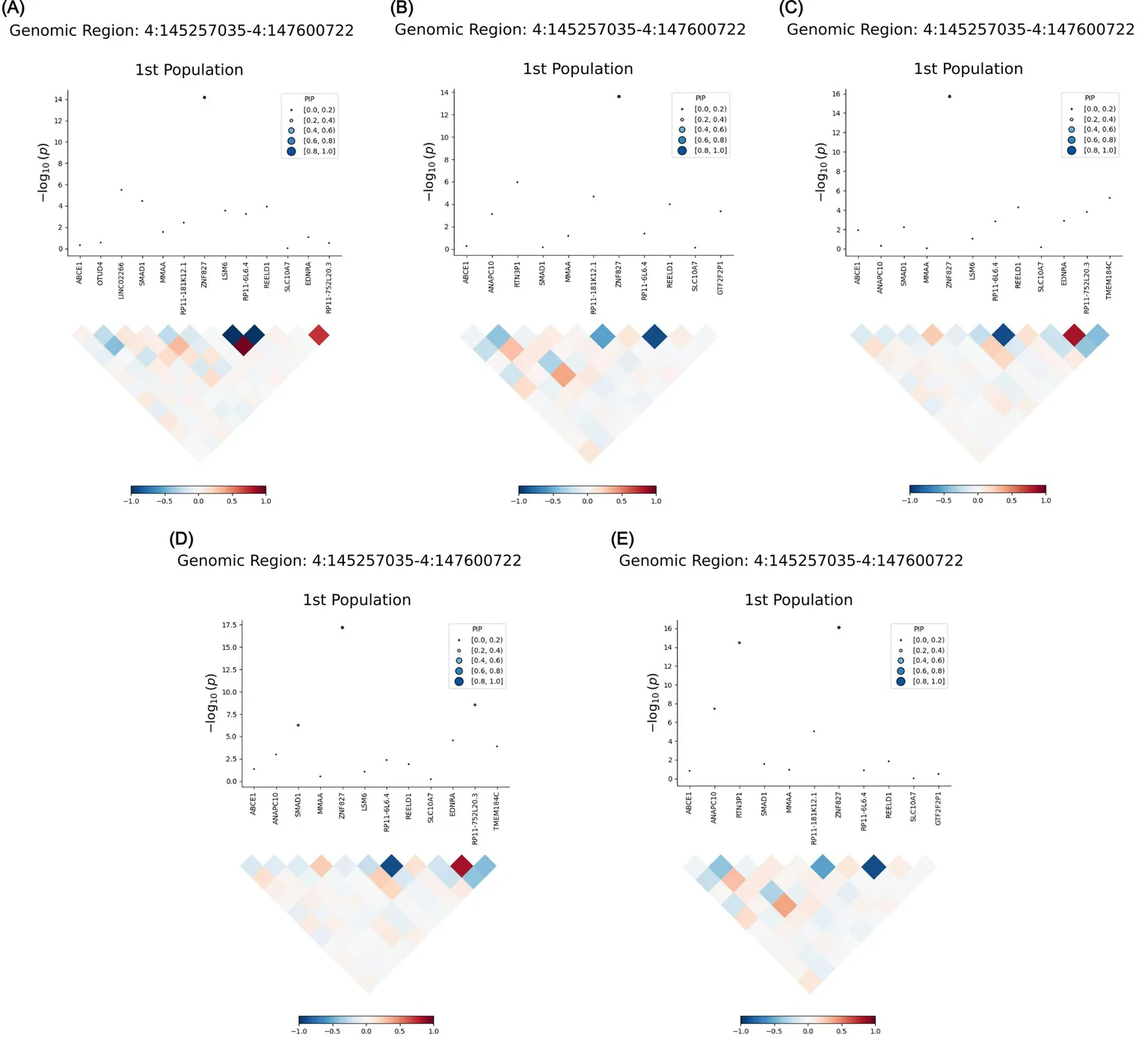
The result of precise localization of chromosome 4 region (145257035–147,600,722) using FOCUS software. **(A–C)** AA analysis: The significant gene *ZNF827* was identified in Adipose Visceral Omentum **(A)**, Artery Tibial **(B)**, and Artery Aorta **(C)** tissues. **(D,E)** TAA analysis: The significant gene *ZNF827* was also identified in Artery Tibial **(D)** and Artery Aorta **(E)** tissues.

For AA, the identified genes included the ADH cluster (*ADH1A, ADH1B, ADH4*) and *ZNF827*, with causal contributions in adipose, arterial, and brain tissues. The three ADH genes co-localized within a narrow locus on chromosome 4 (98,502,916–99,757,203), suggesting shared regulatory mechanisms. *ZNF827* showed exceptionally high PIP values in arterial tissues (aorta, tibial artery), robustly supporting its causal role in AA pathogenesis (Figures 6A–C).

For AAA, fine-mapping implicated only two core genes (*WDR12* and *ICA1L*) distributed across eight tissue types: amygdala, hypothalamus, pancreas, and five others. Notably, *ICA1L* demonstrated multi-tissue associations in peripheral systems, particularly in fibroblasts and gastroesophageal junction tissues (Supplementary Table S23). No involvement of ADH genes or *ZNF827* was detected in AAA.

For TAA, two key genes were identified: *ZNF827* (in five tissues) and *EDNRA* (in three tissues), distributed across eight tissue types including aorta, tibial artery, and liver. *ZNF827* showed maximal causal evidence in vascular tissues (aorta, tibial artery) and gastrointestinal sites, consistent with its pattern in AA (Figures 6D,E).

### 3.6 GO and KEGG analysis

**Figure 7:**
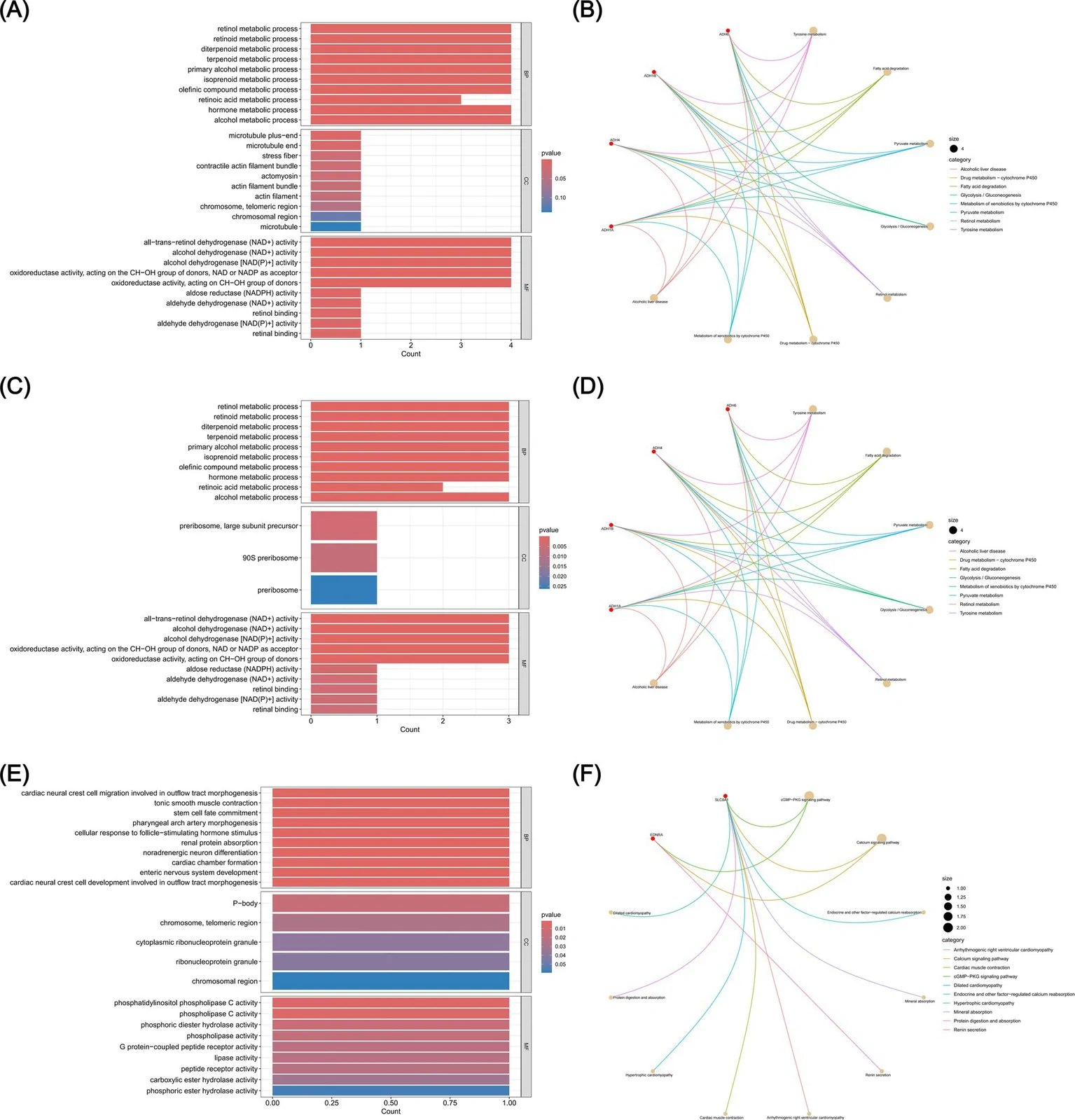
GO and KEGG enrichment analysis for AA, AAA, and TAA. **(A,C,E)** Bar plot representing the GO analysis results for AA, AAA, and TAA. **(B,D,F)** Network diagram representing the KEGG analysis results for AA, AAA, and TAA.

Functional enrichment analyses (GO and KEGG) were performed on gene sets associated with AA (7 genes), AAA (6 genes), and TAA (4 genes), revealing distinct yet overlapping biological insights. GO annotation for both AA and AAA genes showed significant enrichment in similar biological processes (BP), primarily retinol and diterpenoid metabolic processes, while their molecular functions (MF) were both enriched for all-trans-retinol dehydrogenase (NAD+) activity and alcohol dehydrogenase (NAD+) activity. However, their cellular components (CC) differed: AA genes were associated with microtubule plus-ends, stress fibers, and contractile actin filament bundles, whereas AAA genes were linked to ribosomal biogenesis structures like the preribosome, large subunit precursor, and 90S preribosome (Supplementary Table S24; Figures 7A,C). KEGG pathway analysis further highlighted this AA/AAA similarity, showing both sets were primarily involved in tyrosine metabolism, fatty acid degradation, and pyruvate metabolism (Supplementary Table S25; Figures 7B,D) In contrast, TAA-associated genes exhibited markedly different enrichment patterns. Their GO BP terms pointed towards developmental and contractile processes like cardiac neural crest cell migration involved in outflow tract morphogenesis, tonic smooth muscle contraction, and stem cell fate commitment. Their CC included the P-body, chromosome, and telomeric region, and their MF were enriched for enzymatic activities such as phosphatidylinositol phospholipase C activity, phospholipase C activity, and phosphoric diester hydrolase activity (Supplementary Table S24; Figure 7E) Correspondingly, KEGG analysis for TAA genes revealed involvement in distinct pathways including renin secretion and vascular smooth muscle contraction, underscoring the unique functional profile of these genes compared to those associated with AA and AAA (Supplementary Table S25; Figure 7F).

### 3.7 PPI network analysis

Based on PPI network analysis, we constructed core gene interaction networks for AA and its subtypes (AAA and TAA) (Supplementary Figures S1–S3). Notably, ADH family members (*ADH1A/ADH1B/ADH4/ADH6*) were enriched in both AA and AAA networks, while *ZNF827* showed cross-subtype sharing between AA and TAA networks. These subtype-specific networks reveal distinct molecular interaction modules, providing a structural framework for mechanistic studies.

### 3.8 Validation set

We externally validated candidate genes identified in the FinnGen R12 cohort. In the UK Biobank, only *ADH6* among the seven AA-associated candidates retained significance. For AAA, both *ADH6* and *WDR12* demonstrated consistent associations in the UK Biobank. However, none of the TAA-associated genes were validated in the Roychowdhury et al. GWAS dataset, suggesting limited generalizability of the findings for TAA in this validation cohort. Detailed validation statistics from UTMOST, FUSION, and MAGMA analyses are provided in Supplementary Tables S26–S28.

### 3.9 PheWAS analysis

To further evaluate off-target effects and pleiotropy of drug target genes, we conducted a PheWAS at the genetic level. As detailed in Supplementary Figures S4–S25, none of the 11 candidate drug targets for AA and its subtypes (AAA and TAA) exhibited associations with other traits reaching genome-wide significance (*p* < 5 × 10^−8^). This absence of significant genetic-level associations implies limited off-target effects and horizontal pleiotropy, thereby bolstering confidence in the robustness of our study’s findings.

### 3.10 RT-qPCR

**Figure 8:**
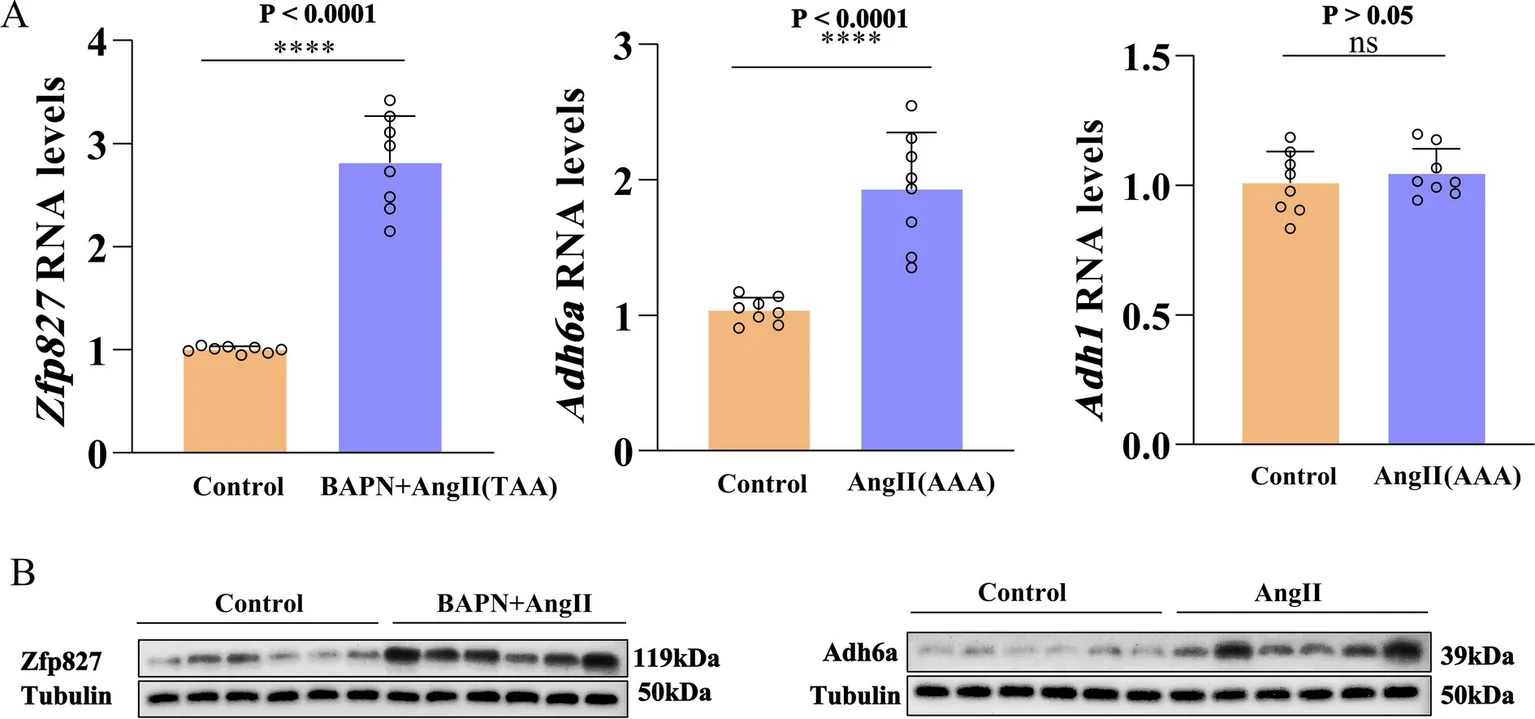
Validation of candidate gene expression in mouse models of TAA and AAA. **(A)** RT-qPCR analysis of *Zfp827, Adh6a*, and *Adh1* mRNA levels in aortic tissues from TAA and AAA model mice compared with controls. Data are presented as mean ± SEM. **(B)** Western blot analysis of *Zfp827* and *Adh6a* protein expression in aortic tissues from TAA and AAA model mice. Tubulin was used as a loading control.

We validated the aortic expression of key candidate genes identified by cross-tissue TWAS using RT-qPCR in mouse models of TAA and AAA. In the TAA model, the expression of *Zfp827*, the murine ortholog of human *ZNF827*, was significantly upregulated compared with controls (*p* < 0.0001), supporting its potential role in transcriptional regulation during TAA pathogenesis. Similarly, in the AAA model, *Adh6a*, the murine homolog of human *ADH6*, showed a significant increase in aortic expression (*p* < 0.0001), consistent with its prior genetic association signal. In contrast, *Adh1*, the murine counterpart of human *ADH1A,* exhibited no significant differential expression in aortic tissue, despite its genetic association with AAA identified by Mendelian randomization analysis (Figure 8A). This finding suggests that *ADH1A* may influence AAA risk through indirect or systemic mechanisms rather than via local transcriptional regulation in the aorta. Primer sequences are listed in Supplementary Table S29.

### 3.11 Western blot

To validate these findings at the protein level, Western blot analysis was performed on aortic tissues from the same mouse cohorts. In the TAA model, *Zfp827* protein expression was significantly elevated compared with controls, consistent with the observed upregulation at the mRNA level. This concordance suggests a potential role for *Zfp827* in the pathogenesis of TAA. Similarly, in the AAA model, *Adh6a* protein expression was significantly increased, supporting its involvement in AAA pathogenesis (Figure 8B).

## 4 Discussion

AA remains a life-threatening vascular disorder with poorly understood molecular mechanisms. While GWAS have identified numerous risk loci for AA and its subtypes (AAA and TAA), the functional interpretation of non-coding variants and their tissue-specific regulatory mechanisms remain challenging. TWAS have emerged as a powerful tool to bridge this gap by integrating gene expression and genetic data. However, most existing TWAS efforts in AA have been limited to single-tissue analyses, which may fail to capture the systemic nature of the disease. Leveraging GWAS summary statistics from the FinnGen R12 cohort and multi-tissue eQTL data from GTEx V8, we conducted a comprehensive cross-tissue TWAS to elucidate the genetic basis of AA and its subtypes (AAA and TAA). By integrating multiple analytical approaches —including cross-tissue and single-tissue TWAS, gene-based MAGMA analysis, MR, colocalization, metabolite mediation, fine-mapping, and functional enrichment—we identified several novel susceptibility genes, notably members of the ADH gene family (*ADH1A, ADH1B, ADH4, ADH6*), *ZNF827*, *ICA1L*, *WDR12*, *EDNRA*, *SLC8A1*, and *TNRC6B*. These findings not only enhance our understanding of the genetic architecture of AA and its subtypes (AAA and TAA)but also reveal potential mechanistic pathways underlying its pathogenesis.

Our study introduces a key novelty: integrating multi-omics layers (genetic, cross-tissue transcriptomic, and metabolomic) within a single analytical framework, combined with experimental validation in animal models. While prior TWAS on AA have largely focused on single-tissue predictions or independent gene discovery, our approach uniquely combines cross-tissue TWAS with metabolite-mediated Mendelian randomization and *in vivo* validation. This design enables us to move beyond mere association toward identifying causal metabolic pathways that link genetic variants to disease risk.

A key finding of our study is the identification of *ZNF827* as a cross-subtype risk gene significantly associated with both AA and TAA. *ZNF827,* located on chromosome 4, encodes a zinc finger protein that has been implicated in DNA damage response and chromatin regulation (35–37). Our colocalization and FOCUS fine-mapping robustly support its causal role in vascular tissues, suggesting that *ZNF827* may influence aortic wall integrity through regulation of vascular smooth muscle cell function, extracellular matrix remodeling, or inflammatory responses. Functionally, *ZNF827* acts as a single-stranded DNA-binding protein that facilitates homologous recombination repair during replication stress via its C2H2 zinc finger domain and interaction with replication protein A, thereby participating in the ATR–CHK1 DNA damage response pathway (38). Furthermore, *ZNF827* contributes to epigenetic regulation by recruiting histone deacetylase 1, influencing chromatin organization and RNA splicing, with established roles in epithelial–mesenchymal transition, cancer metastasis, and brain development (39). In the context of tissue integrity and stress response, *ZNF827* has also been identified as a risk locus in alcohol-associated liver disease, particularly among non- and light drinkers, underscoring its broader role in cellular stress adaptation (40). Although direct mechanistic evidence in AA and its subtypes (AAA and TAA) remains limited, *ZNF827*’s central functions in DNA repair, chromatin remodeling, and tissue homeostasis suggest plausible contributions to AAA and TAA pathogenesis via vascular cell dysfunction, matrix destabilization, or inflammatory activation. Future studies should focus on delineating the expression and functional role of *ZNF827* within vascular cells to clarify its impact on aortic wall integrity.

Equally noteworthy is the consistent association of the ADH gene family (*ADH1A*, *ADH1B*, *ADH4*, *ADH6*) with AA and AAA. Located at chromosome 4, this gene family encodes enzymes essential for metabolizing ethanol, retinol, and other endogenous substrates (41). These enzymes catalyze retinol oxidation to retinaldehyde, a rate-limiting step in retinoic acid (RA) synthesis (42, 43). RA serves as a key signaling molecule that regulates cell differentiation, immune responses, and vascular homeostasis. In adults, RA signaling maintains vascular homeostasis by modulating vascular smooth muscle cell phenotypes, inhibiting proliferation, and regulating inflammatory responses (44). Recent studies have found that mutations in the *ADH1B* gene are significantly associated with non-alcoholic fatty liver disease (NAFLD) (45). *ADH1B* influences hepatic lipid accumulation and NAFLD progression through ethanol and retinol metabolism. Similarly, another study found that the *ADH1B*-rs1229984 variant influences alcohol metabolism and associates with cirrhosis, stroke, gout, and esophageal cancer risks (46). Our multi-method analysis consistently identified *ADH1A, ADH1B, ADH4,* and *ADH6* as significantly associated with AA and AAA risk. Mediation analysis reveals that N-acetylphenylalanine and methionine sulfoxide partially mediate *ADH1A/ADH6* effects, suggesting a metabolite-immune axis in AA pathogenesis. This aligns with emerging evidence that dysregulated immunometabolism plays a key role in aortic remodeling. Importantly, the ADH cluster co-localizes within a narrow genomic region on chromosome 4, indicating shared regulatory elements and possibly coordinated expression.

From a nutritional and metabolic perspective, several aspects of our findings warrant emphasis. First, although the ADH family’s role in retinol oxidation is well recognized, genetic variation in these genes may alter vitamin A metabolic flux, thereby modulating AA susceptibility through a nutritionally modifiable pathway. Second, our metabolite mediation analysis directly implicates diet-derived compounds not previously linked to AA: N-acetylphenylalanine (a gut microbial metabolite of dietary phenylalanine), methionine sulfoxide (an oxidized product of the essential amino acid methionine), and campesterol—a plant sterol abundant in vegetable oils and margarine. The mediation of *ADH6* effects by campesterol in AAA suggests a potential link between dietary phytosterol intake and AAA risk. Third, the enrichment in fatty acid degradation and pyruvate metabolism pathways highlights how systemic energy metabolism—influenced by diet and nutritional state—may differentially impact AA versus TAA. Collectively, these findings position our genetic discoveries within a testable nutritional framework, opening avenues for future dietary intervention studies.

Functional enrichment analysis revealed distinct biological pathways for AA/AAA and TAA. While AA and AAA were enriched in metabolic pathways (e.g., retinol and fatty acid degradation), TAA-associated genes were involved in developmental and contractile processes (e.g., neural crest migration and smooth muscle contraction). These findings underscore the distinct etiologies between AA/AAA and TAA, which may necessitate different therapeutic strategies. For instance, interventions targeting metabolic pathways may be more effective for AA and AAA, whereas modulators of developmental signaling may be relevant for TAA.

Our study also advances the field methodologically. The use of FOCUS fine-mapping provided tissue-specific causal probability estimates, highlighting high-confidence genes like *ZNF827* and ADH family members. Moreover, the PheWAS analysis showed no significant off-target effects for the prioritized genes, suggesting they may be promising and specific therapeutic targets. The integration of metabolomic mediation analysis further provided mechanistic hypotheses linking genetic variants to disease via metabolic pathways.

Despite these strengths, several limitations should be acknowledged. First, the predominantly European ancestry of our study cohort may limit the generalizability of findings to other populations. Future studies in diverse populations and with larger sample sizes are needed. Second, despite employing multiple methods to minimize false positives, the absence of an independent replication cohort for certain subtypes (e.g., TAA) represents a methodological limitation. Third, while our study provides initial *in vivo* validation of key candidates like *ZNF827* and *ADH6* in mouse models, more detailed functional validation of identified genes and metabolites in relevant human vascular tissues or cell types (e.g., aortic smooth muscle cells) remains an important direction for future investigation.

## 5 Conclusion

In summary, our cross-tissue TWAS coupled with experimental validation delivers a comprehensive and integrated genetic landscape of AA and its subtypes (AAA and TAA). We have identified novel susceptibility genes and provided initial functional support for *ZNF827* and *ADH6*, thereby advancing the mechanistic understanding of AA and its subtypes (AAA and TAA) pathogenesis. These findings not only provide deeper insights into the molecular underpinnings of AA and its subtypes but also present promising therapeutic targets. Crucially, by identifying specific nutrient-related metabolites (e.g., phytosterols, amino acid derivatives) and metabolic pathways (e.g., retinol metabolism) as mediators of genetic risk, our study lays the groundwork for future nutritional and metabolic intervention studies. This underscore the necessity of developing subtype-specific treatment strategies and highlight the potential role of dietary and metabolic modulators in future research.

## Data Availability

The original contributions presented in the study are included in the article/Supplementary material, further inquiries can be directed to the corresponding author.

## Footnotes

1
https://ftp.ebi.ac.uk/pub/databases/spot/eQTL/imported/GTEx_V8

2
https://www.finngen.fi/en/access_results

3
https://github.com/Joker-Jerome/UTMOST?tab=readme-ov-file

4
http://gusevlab.org/projects/fusion/

5
https://cnsgenomics.com/software/smr/#Overview

6
http://genemania.org

7
https://pan.ukbb.broadinstitute.org

8
https://azphewas.com/
