# Supplementary material for: Integrative cross-tissue transcriptome-wide association and metabolomic analysis reveals novel genetic risk loci for aortic aneurysm
Source: Front Nutr. 2026 Aug 24;13:1795141. doi: 10.3389/fnut.2026.1795141 (PMC13547935; doi:10.3389/fnut.2026.1795141)
Supplement: Supplementary file 1 [file Supplementary_file_1.DOCX]

Figure S1-3. PPI network analysis.

Figure S1. PPI network constructed using GeneMANIA for AA.

Figure S2. PPI network constructed using GeneMANIA for AAA.

Figure S3. PPI network constructed using GeneMANIA for TAA.

Figure S4-25. PheWAS analysis of genes related to subtypes of aortic aneurysm (AA, TAA, AAA).

Figure S4. Binary traits PheWAS association with ADH1A

Figure S5. Continuous traits PheWAS association with ADH1A

Figure S6. Binary traits PheWAS association with ADH1B

Figure S7. Continuous traits PheWAS association with ADH1B

Figure S8. Binary traits PheWAS association with ADH4

Figure S9. Continuous traits PheWAS association with ADH4

Figure S10. Binary traits PheWAS association with ADH6

Figure S11. Continuous traits PheWAS association with ADH6

Figure S12. Binary traits PheWAS association with CCDC74A

Figure S13. Continuous traits PheWAS association with CCDC74A

Figure S14. Binary traits PheWAS association with EDNRA

Figure S15. Continuous traits PheWAS association with EDNRA

Figure S16. Binary traits PheWAS association with GAS2L1

Figure S17. Continuous traits PheWAS association with GAS2L1

Figure S18. Binary traits PheWAS association with ICA1L

Figure S19. Continuous traits PheWAS association with ICA1L

Figure S20. Binary traits PheWAS association with TNRC6B

Figure S21. Continuous traits PheWAS association with TNRC6B

Figure S22. Binary traits PheWAS association with WDR12

Figure S23. Continuous traits PheWAS association with WDR12

Figure S24. Binary traits PheWAS association with ZNF827

Figure S25. Continuous traits PheWAS association with ZNF827
